# Effects of immune inflammation in head and neck squamous cell carcinoma: Tumor microenvironment, drug resistance, and clinical outcomes

**DOI:** 10.3389/fgene.2022.1085700

**Published:** 2022-12-12

**Authors:** Li Zhu, Yue Wang, Xingzhong Yuan, Yifei Ma, Tian Zhang, Fangwei Zhou, Guodong Yu

**Affiliations:** ^1^ Department of Otorhinolaryngology Head and Neck Surgery, Affiliated Hospital of Guizhou Medical University, Guiyang, China; ^2^ Department of Clinical Medicine, Guizhou Medical University, Guiyang, China

**Keywords:** HNSCC, inflammatory factor-related gene, immune pathway, prognosis, bioinformatics

## Abstract

**Background**: Head and neck squamous cell carcinoma (HNSCC) is a malignant tumor with a very high mortality rate, and a large number of studies have confirmed the correlation between inflammation and malignant tumors and the involvement of inflammation-related regulators in the progression of HNSCC. However, a prognostic model for HNSCC based on genes involved in inflammatory factors has not been established.

**Methods**: First, we downloaded transcriptome data and clinical information from patients with head and neck squamous cell carcinoma from TCGA and GEO (GSE41613) for data analysis, model construction, and differential gene expression analysis, respectively. Genes associated with inflammatory factors were screened from published papers and intersected with differentially expressed genes to identify differentially expressed inflammatory factor-related genes. Subgroups were then typed according to differentially expressed inflammatory factor-related genes. Univariate, LASSO and multivariate Cox regression algorithms were subsequently applied to identify prognostic genes associated with inflammatory factors and to construct prognostic prediction models. The predictive performance of the model was evaluated by Kaplan-Meier survival analysis and receiver operating characteristic curve (ROC). Subsequently, we analyzed differences in immune composition between patients in the high and low risk groups by immune infiltration. The correlation between model genes and drug sensitivity (GSDC and CTRP) was also analyzed based on the GSCALite database. Finally, we examined the expression of prognostic genes in pathological tissues, verifying that these genes can be used to predict prognosis.

**Results**: Using univariate, LASSO, and multivariate cox regression analyses, we developed a prognostic risk model for HNSCC based on 13 genes associated with inflammatory factors (ITGA5, OLR1, CCL5, CXCL8, IL1A, SLC7A2, SCN1B, RGS16, TNFRSF9, PDE4B, NPFFR2, OSM, ROS1). Overall survival (OS) of HNSCC patients in the low-risk group was significantly better than that in the high-risk group in both the training and validation sets. By clustering, we identified three molecular subtypes of HNSCC carcinoma (C1, C2, and C3), with C1 subtype having significantly better OS than C2 and C3 subtypes. ROC analysis suggests that our model has precise predictive power for patients with HNSCC. Enrichment analysis showed that the high-risk and low-risk groups showed strong immune function differences. CIBERSORT immune infiltration score showed that 25 related and differentially expressed inflammatory factor genes were all associated with immune function. As the risk score increases, specific immune function activation decreases in tumor tissue, which is associated with poor prognosis. We also screened for susceptibility between the high-risk and low-risk groups and showed that patients in the high-risk group were more sensitive to talazoparib-1259, camptothecin-1003, vincristine-1818, Azd5991-1720, Teniposide-1809, and Nutlin-3a (-) −1047.Finally, we examined the expression of OLR1, SCN1B, and PDE4B genes in HNSCC pathological tissues and validated that these genes could be used to predict the prognosis of HNSCC.

**Conclusion**: In this experiment, we propose a prognostic model for HNSCC based on inflammation-related factors. It is a non-invasive genomic characterization prediction method that has shown satisfactory and effective performance in predicting patient survival outcomes and treatment response. More interdisciplinary areas combining medicine and electronics will be explored in the future.

## 1 Introduction

Head and neck squamous cell carcinoma (HNSCC) is a tumor located above the clavicle and below the base of the skull. It includes tumors of the neck, otorhinopharynx, and the oral and maxillofacial regions. Squamous cell carcinoma accounts for approximately 90% of all head and neck malignancies (Thompson and Franchi, 2018). HNSCC is the sixth most common malignancy worldwide, with over 930,000 new cases and 460,000 deaths reported in 2020 (Sung et al., 2021). In the United States, head and neck cancer accounts for 3% of all malignancies and more than 1.5% of deaths (Mourad et al., 2017). It is the most common type of head and neck tumor (up to 90%) (Rahman et al., 2019). Furthermore, its prognosis is poor (Pi et al., 2017) with a general 5-year survival rate of less than 40% and a 5-year survival rate of only 39.1% in patients with metastases (Anand et al., 2021; Muzaffar et al., 2021). Previous etiological studies suggested that smoking and alcohol use are common risk factors (Leemans et al., 2018). Despite improvements in standard treatments, supportive care, overall survival (OS) times, and the quality of life for patients, the prognosis for HNSCC remains poor with a global 5-year survival rate of approximately 50% as of 2011. Therefore, studying the molecular mechanisms underlying HNSCC development has important clinical implications for the exploration of more effective treatment strategies.

Inflammation is the “seventh hallmark of cancer” (Bonomi et al., 2014) and many studies have shown that it plays a vital role in the development and progression of cancer (Kris et al., 2014; Man and Kanneganti, 2015). The role of inflammation in the development and progression of cancer has been a significant focus of cancer research since the relationship between inflammation and cancer was first reported in 1863 (Wang et al., 2009; Khandia and Munjal, 2020). The relationship between tumors and inflammatory responses can be reflected by the levels of specific substances in blood or tumor specimens (Allen et al., 2007), including interleukin-6, IL-8, growth-associated oncogene -1, vascular endothelial growth factor, hepatocyte growth factor, and cytokines, and elevated levels of growth factors associated with tumor progression and recurrence (Karki et al., 2017). Whereas these cytokines may lead to co-occurring immune stimulation and immunosuppression in cancer patients, concentrations of cytokines MIF, TNFα, interleukin 6, interleukin 8, interleukin 10, interleukin 18, and TGFβ are increased (Lippitz, 2013; Wang et al., 2017). This specific cytokine pattern appears to have a prognostic effect, as high interleukin 6 or interleukin 10 serum concentrations are associated with poor prognosis in independent cancer types (Lippitz, 2013). Although immunostimulatory cytokines are involved in local cancer-related inflammation, cancer cells appear to be under cytokine-mediated local immunosuppression (Miller et al., 1994). Inflammatory cytokines produced by tumors may play a critical role in this immunodeficiency. Further studies have shown that the polymorphic expression of inflammation-related genes (IRGs) is associated with the development of squamous cell carcinoma of the nasopharynx (Brunotto et al., 2014). This suggests that IRGs play an essential role in nasopharyngeal carcinoma development. As a secondary role, the tumor microenvironment is also associated with tumor promotion and progression, which may be related to IRGs, and to some extent, reflected in immune cells. In recent years, rapid advances in sequencing technology and statistics have enabled researchers to investigate the role of signature genes in cancer prognosis (Ren et al., 2021; Yuan et al., 2022a). Increasing studies have shown that IRGs play an important role in specific cancer prognosis, such as hepatocellular carcinoma and colon cancer (17, 18). However, few studies have used IRGs as prognostic markers in head and neck squamous cell carcinoma (Liang et al., 2021; Lin et al., 2021). However, few studies have used IRGs as prognostic markers in nasopharyngeal carcinoma.

This study aimed to develop a transcriptomics-based approach to reveal the immune cell activation status and predict the survival outcomes of patients with HNSCC. We collected two sets of transcriptional profiling data and corresponding clinical information from the cancer genome atlas (TCGA) and GEO databases, obtained differential genes based on expression level, explored the level of immune cell activation in HNSCCs, and constructed a prognostic model for HNSCC. We also identified several differential genes associated with immune activation as potential biomarkers. Additionally, we performed a comprehensive analysis of the risk model, including functional enrichment, immune activation, and immune infiltration. Our findings reveal the critical role played by immune activation in HNSCC and we propose a convenient approach to help diagnose and predict survival outcomes in patients with HNSCC.

## 2 Materials and methods

**FIGURE 1:**
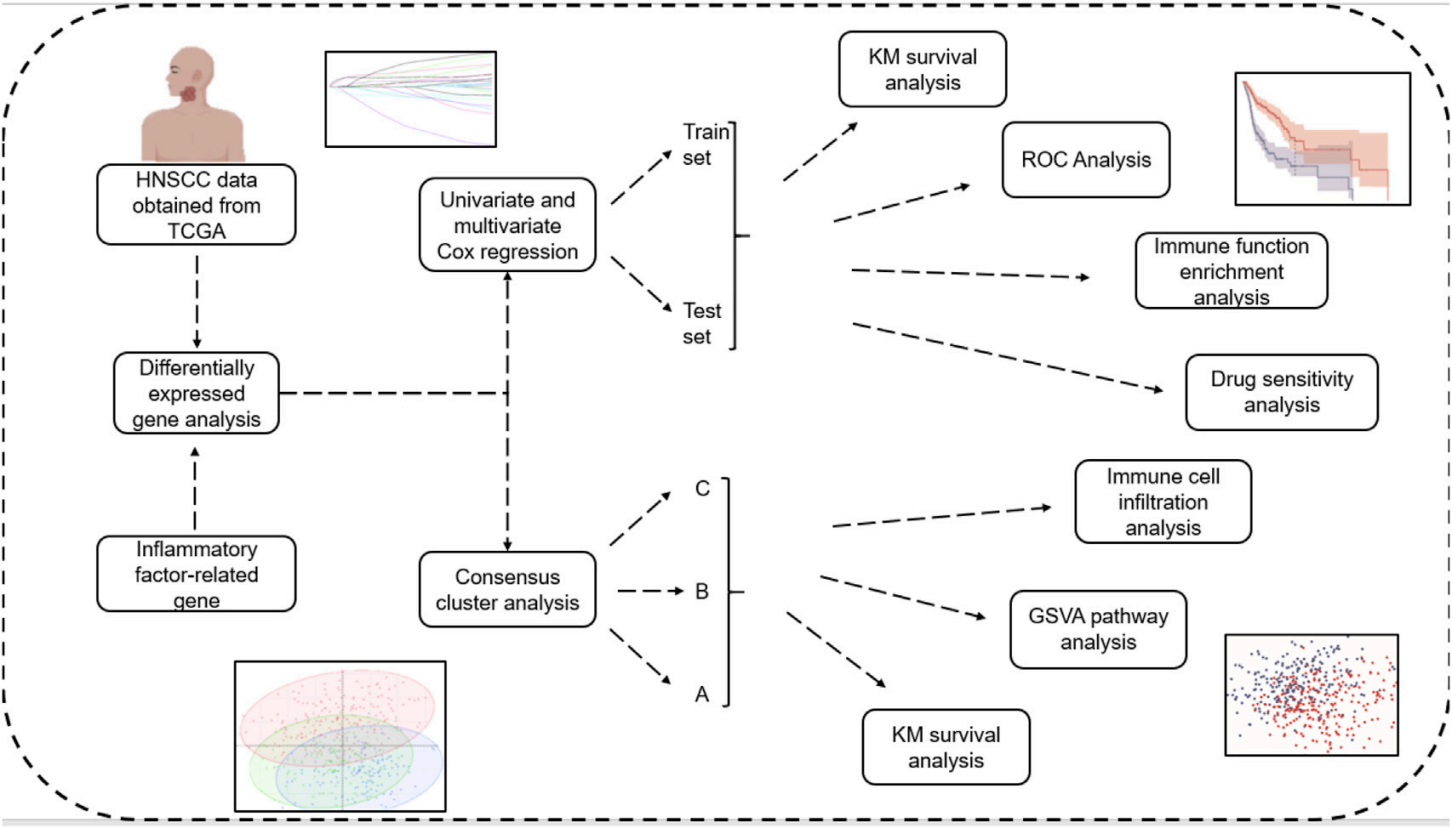
Flowchart.

The flow chart of this article was shown in Figure 1.

### 2.1 Data sources

Gene expression data from TCGA database were downloaded from 505 patients with HNSCC and there were 44 partially matched paracancerous tissue samples. Clinical data for the HNSCC samples, including age, sex, survival time, survival status, tumor stage, and TNM staging, were also downloaded. The microarray dataset GSE41613, which explores the gene expression profile of human HNSCC, was obtained by searching the GEO (https://www.ncbi.nlm.nih.gov/geo/) database for “HNSCC.” The GSE41613 microarray data, which is based on the GPL570 platform, contained 98 HNSCC patients that were subsequently used for external validation of the model. A total of 198 inflammatory response-related genes (IRGs) were included based on the MSigDB database and previous studies, which were displayed in Supplementary Table S1. The data were preprocessed as follows: the probes were corresponded to the genes, null probes were removed, and multiple probes corresponded to the same gene. Then, the median gene expression level was selected.

### 2.2 Extraction of relevant differentially expressed genes (DEGs)

Because different data came from different platforms, PCA plots before and after batch effect elimination were performed using each data from the ComBat method in the sva package to eliminate batch effects. Differential analysis between tumor samples and standard samples from the TCGA-HNSCC dataset was performed using the R package edgeR. The limma package in R was used to screen DEGs. Screening criteria were abs | logFC | > 0.1 and *p* < 0.05. Venn diagrams were drawn by intersecting differentially expressed genes with genes involved in inflammatory responses *via* the Rvenn “package. To visualize gene expression, a reciprocal network of differentially expressed IRGs was constructed using the GeneMania database to draw a heat map of differential expression. Differentially expressed IRGs were subsequently subjected to univariate, LASSO and multivariate Cox regression analysis for model construction.

### 2.3 Consensus clustering of subtypes based on differentially expressed IRGs

We performed non-negative matrix factorization (NMF) clustering analysis to develop molecular subtypes based on differentially expressed IRGs expression profiles. For the NMF method, the standard “brunet” option was selected and 10 iterations were performed. The number of clusters was set from 2 to 9, and the average profile width of the common membership matrix was determined by the R package ‘NMF’ with a minimum membership of 10 for each subclass. The optimal number of clusters was determined to be 3 by co-occurrence, dispersion and contour indices. Heat maps, principal component analysis (PCA) maps between subgroups, and prognostic KM curves were plotted for the correlation between gene expression levels and clinicopathological characteristics between subcategories.

### 2.4 Cluster analysis

The “c2.cp.kegg.v7.5.1. symbols” gene set was downloaded from the MSigDB database and a gene set variation analysis (GSVA) was performed between subgroups. R package GSVA was used to calculate the scores of the relevant pathways using the single-sample gene set enrichment analysis (ssGSEA) method based on each sample’s gene expression matrix. In addition, the enrichment functions (or pathways) were screened for differences using the limma package. The degree of immune infiltration was also assessed using ssGSEA, and box-line plots were drawn to demonstrate the immune profiles of the different subclasses. Inflammatory factor-related genes selected by univariate Cox regression analysis algorithm were included in multivariate Cox regression, disordered multivariate categorical variables and rank data were set dumb variables, 95% confidence intervals of HR were tested, independent factors affecting prognosis were screened, and LASSO cox regression analysis was used to establish a prognostic model. Patients with TCGA-HNSCC were divided into high-risk and low-risk groups according to the median value area.

### 2.5 Multivariate cox prognostic regression model validation

The PCA plots and t-SNE plots showed that the model differentiated sufficiently between high- and low-risk groups. The KM curves showed significant differences in prognosis between high- and low-risk groups. Then, receiver operating characteristic (ROC) curves were used to assess survival prediction and the area under the ROC curve (AUC) values were calculated using the timeROC R package to measure prognosis or prediction accuracy. The GSE41613 cohort was used as the validation set for model validation and the median risk score was used to differentiate between the high- and low-risk groups.

### 2.6 Enrichment analysis

Gene ontology (GO), Kyoto Encyclopedia of Genes and Genomes (KEGG), GSEA, and GSVA functional enrichment analyses were performed. The GO annotation analysis was performed using R package “clusterProfiler” (version 4.0.5) with a false discovery rate (FDR) < 0.05 to identify significantly enriched pathways. A KEGG enrichment analysis was also performed. GSEA software version 4.1.0 (Broad Institute, Cambridge, MA, United States) was used to identify genomic cohorts that were significantly altered between predefined high- and low-risk groups during consensus clustering. *p*-values <0.05 and an FDR <0.25 were considered statistically significant.

### 2.7 Immuno-infiltration analysis

We uploaded the gene expression matrix data (TPM) to CIBERSORTx for further CIBERSORTx immune infiltration analysis, combined it with the LM22 immune gene set, and filtered the samples with the criterion set at *p* < 0.05 to produce an immune cell infiltration matrix. The correlations between the risk score, essential genes, and the immune cell infiltration level were analyzed. The ESTIMATE R package allows gene expression profiles to predict stromal and immune cell scores and then calculates their values. We analyzed the correlation between the ESTIMATE score and high- and low-risk groups.

### 2.8 Drug sensitivity analysis

The relationship between model genes and drug sensitivity (GSDC and CTRP) was analyzed using the GSCALite database (http://bioinfo.life.hust.edu.cn/GSCA/#/drug) (Figures 7A, B) and drug sensitivity was analyzed using the oncoPredict package to compare drug sensitivity in patients in the high- and low-risk groups.

### 2.9 Organize validation

Five pairs of laryngeal cancer and adjacent tissues from the Affiliated Hospital of Guizhou Medical University, approved by the ethics committee, were used in our investigation. Each participant was informed of the study protocol. None of the patients received other treatments prior to surgery, such as immunotherapy or radiation. Total RNA was extracted from the tissues using TRIzol and stored in liquid nitrogen until required. A Revert Aid First Strand cDNA Synthesis Kit (Thermo Fisher Scientific, Waltham, MA, United States) was used to reverse-transcribe the extracted RNA into cDNA for further analysis. The cDNA concentration was measured to ensure that the required standards were met. GAPDH was used as the internal reference.

### 2.10 Statistics

All statistical analyses were performed in R. The Cox regression analyses were performed using R package survival and survminer for one-way Cox regression and multi-way Cox regression where a *p*-value <0.05 showed that the prognostic variable was significant.

## 3 Results

### 3.1 Identification and correlation analysis of DEGs

**FIGURE 2:**
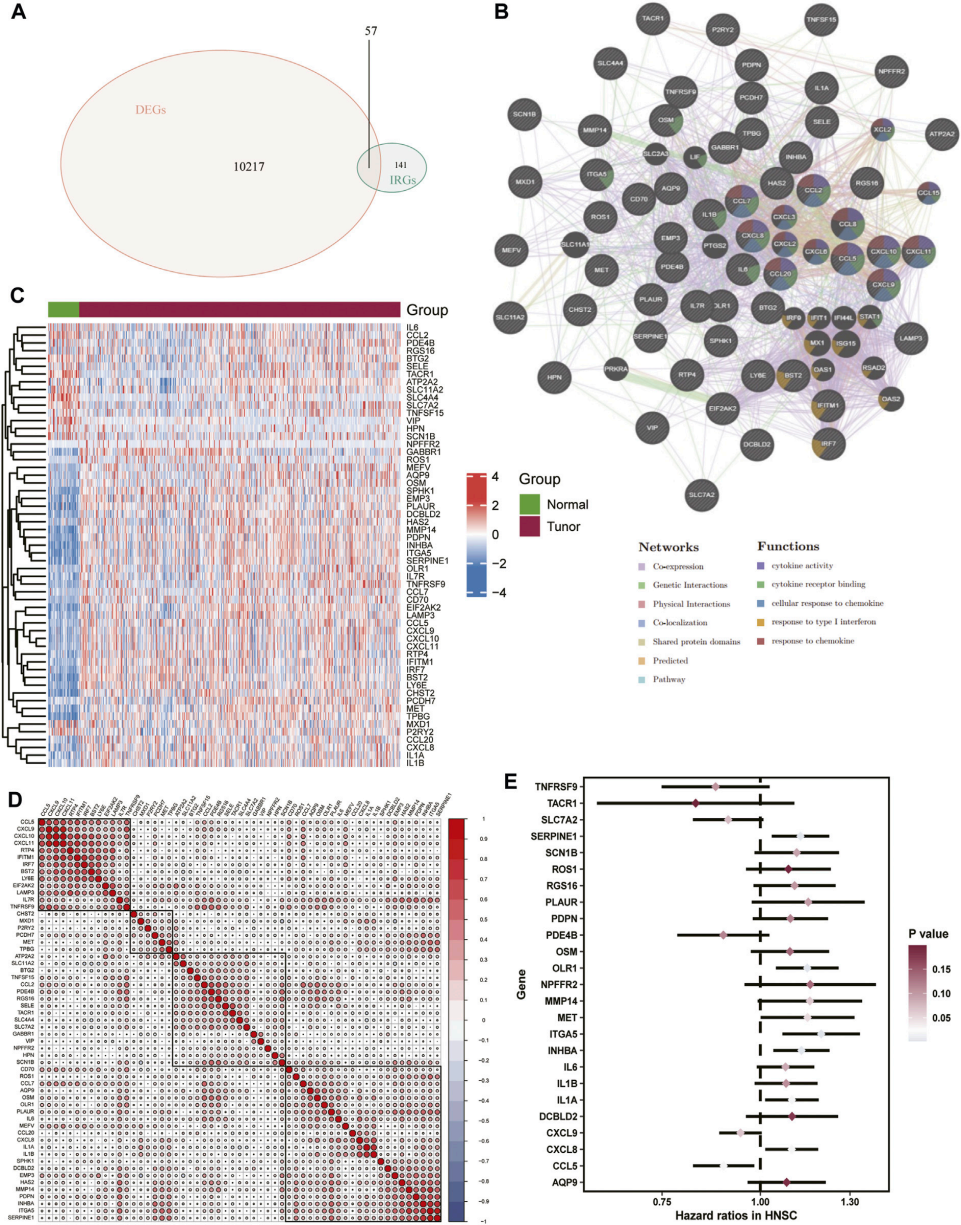
Extraction of correlated DEGs.**(A)** Venn diagram of inflammatory response-related genes *versus* differentially expressed genes in TCGA-HNSC. **(B)** Interaction network map of 57 differentially expressed genes, **(C)** Heat map of differentially expressed genes **(D)** Heat map of correlation between differentially expressed genes. **(E)** Forest plot of the prognostic value of inflammation-associated genes tested.

The DEGs were identified in the skin of HNSCC patients and healthy controls. A total of 10,274 differential genes were obtained from the tumor samples, intersecting 198 inflammatory response-related genes. A reciprocal network for the 57 differentially expressed IRGs obtained (Figure 2A) was mapped using the GeneMania database (Figure 2B), and then, differential expression (Figure 2C) and correlation heat maps (Figure 2D) were plotted for the genes. We further performed a univariate Cox regression analysis on the differentially expressed IRGs and screened 25 inflammatory factor-related genes for prognostic model construction (Figure 2E).

### 3.2 Molecular subtypes associated with inflammation

**FIGURE 3:**
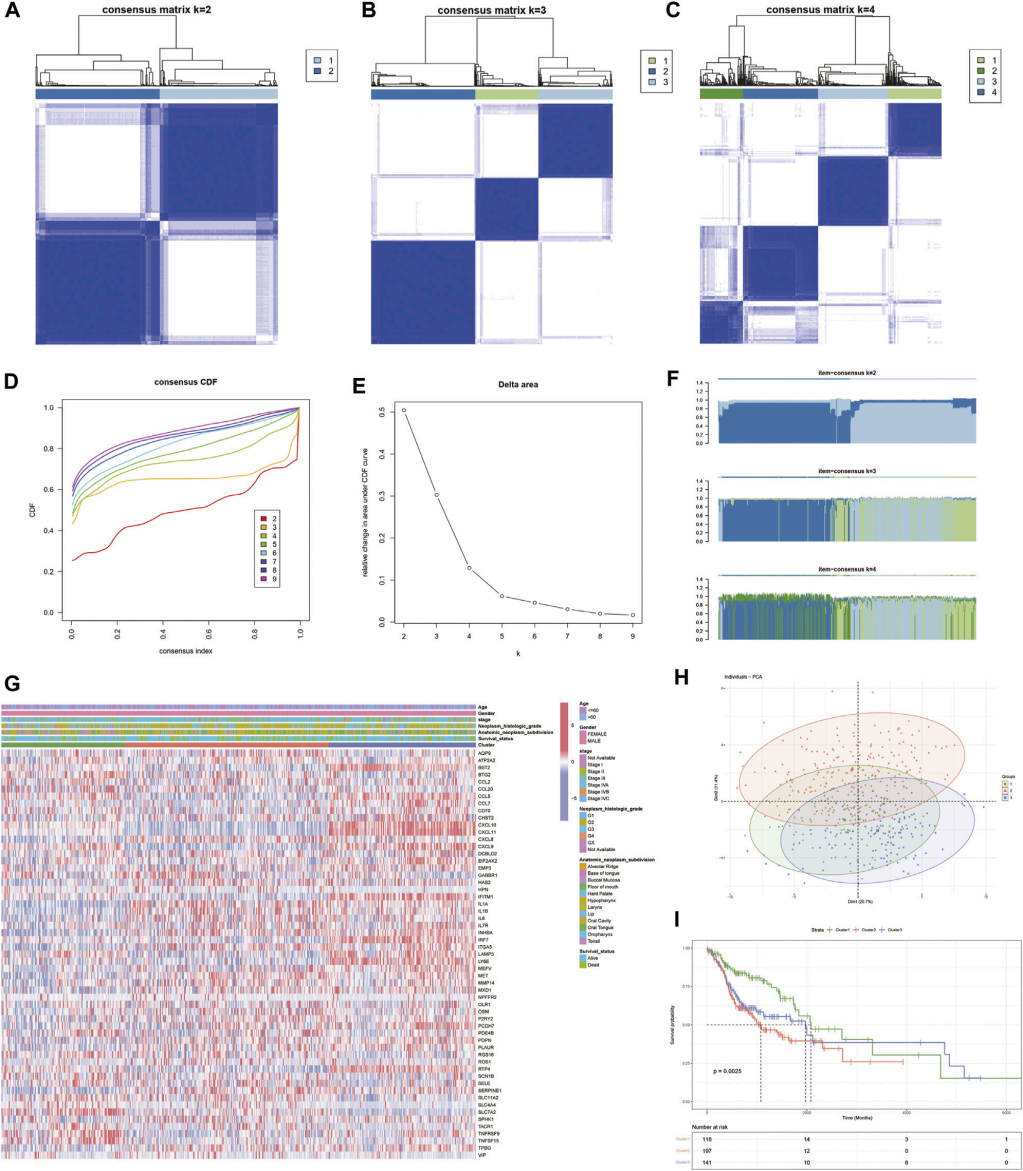
Identification of inflammatory factor gene-associated HNSCC subtypes in the TCGA cohort. **(A–C)** Heat map depicting the consensus matrix at *k* = 2, 3, and 4 in the TCGA cohort. **(D–F)** Cumulative distribution function (CDF), delta area plot and tracling plot in the consensus cluster analysis. **(G)** Shows the association of gene expression levels and clinicopathological features between subclasses. **(H)** PCA plots showing good differentiation. **(I)** Prognostic KM curve plot showing significant differences in prognosis among the three.

An unsupervised consensus clustering analysis based on the 57 differentially expressed IRGs was used to classify 505 patients with HNSCC into different subtypes (*k* = 2, 3, 4, 5, 6, 7, 8, and 9). Molecular typing was then performed on TCGA-HNSCC samples (Figures 3A–C). Three subclasses were identified by the cumulative distribution function (CDF), delta area plot, and tracking plot in the consensus cluster analysis (*k* = 3) as the optimal number of clusters (Figures 3D–F). Heat maps of the association between gene expression levels and the clinicopathological features between subclasses were plotted (Figure 3G). The differences were distinguishable and a PCA analysis was performed to reduce dimensionality and validate the assignment of subtypes. The two-dimensional PCA distribution pattern was confirmed to be consistent with the CDF curve (Figure 3H). This suggested that the two sample groups had been successfully separated. We also explored the differences in survival information between the two groups. The survival curves demonstrated significant differences in OS between the two groups (*p* < 0.0001). Cluster 2 group had a significantly worse prognosis (Figure 3I), suggesting that differentially expressed IRGs can be used to predict patient prognosis.

### 3.3 Construction of the multivariate cox prognostic regression model

**FIGURE 4:**
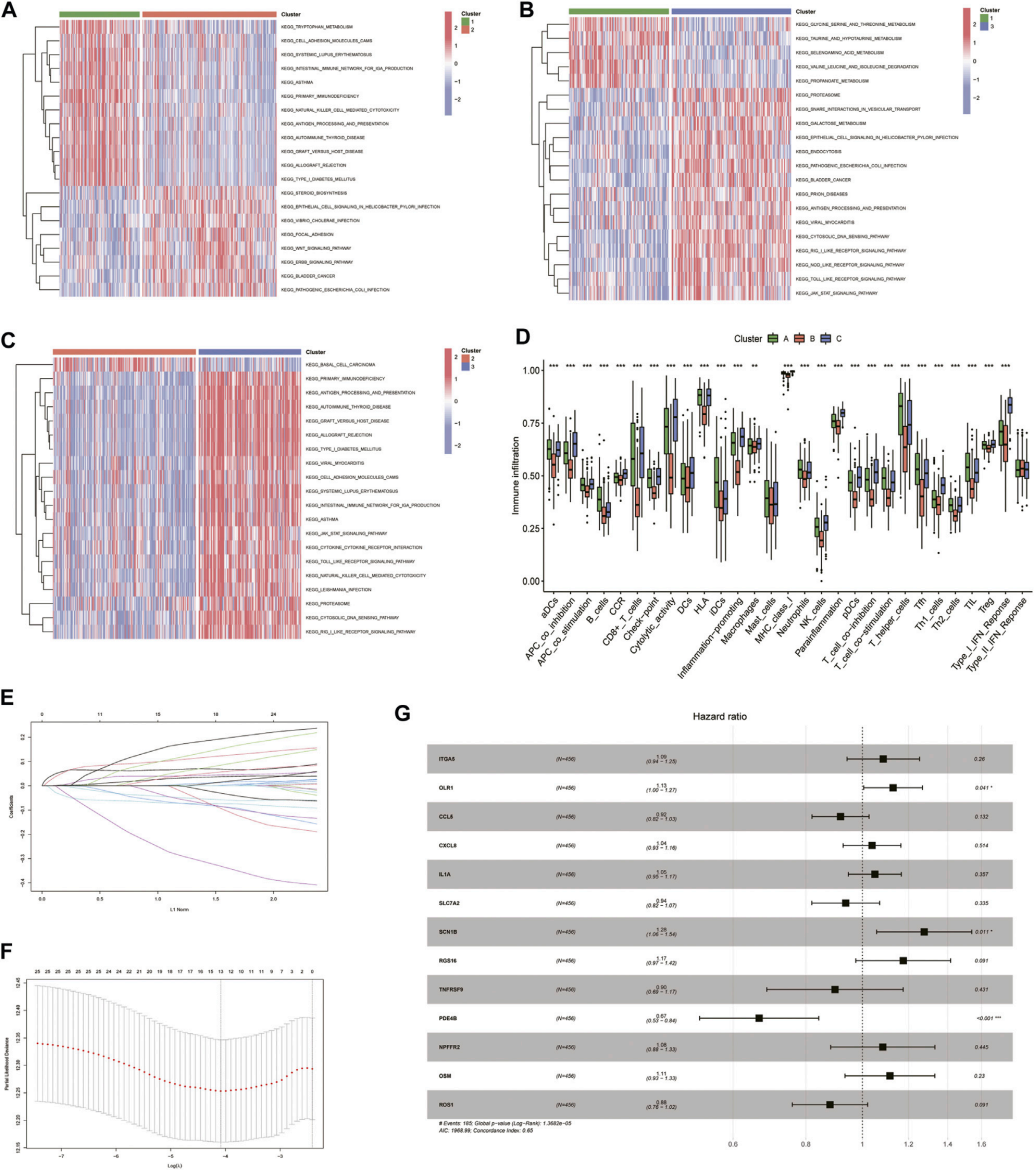
Univariate COX, lasso and multivariate COX regression analysis of overall survival-related inflammation-associated genes using R software. **(A–C)** Heat map showing differences in enrichment function between the 3 subclusters. **(D)** The degree of immune infiltration was assessed using ssGSEA and box-line plots were drawn to demonstrate immunity in different subclasses. **(E)** LASSO regression analysis to calculate the coefficients of inflammation-related genes. **(F)** Thirteen genes were selected as active covariates for cross-validation of LASSO mode. **(G)** Forest plot showing the 13 genes which were selected by stepwise forward and backward regression methods for the Cox proportional hazard model.

The enrichment function results showed that there were significant differences between the three cluster groupings, and these differences were mainly based on immune-related pathways (Figures 4A–C). The degree of immune infiltration was assessed using ssGSEA. Box plots were drawn to demonstrate the immune profile of the different subgroups, and they showed that there was significant variability in both immune cell and immune function activation. Clusters 1 and 3 showed more robust immune function activation than Cluster 2, suggesting that immune function may be prognostically associated with increased immune activation and that it was an improved prognosis method for HNSCC patients (Figure 4D). Based on the 25 genes obtained from the single gene Cox screen (Figures 4E, F), 13 genes were selected from the 25 relevant genes identified by the LASSO regression analysis. A prognostic model was constructed using the following risk score calculation formula: ITGA5*0.0823 + OLR1*0.12103 + CCL5*-0.08642 + CXCL8*0.03798 + IL1A* 0.04907 + SLC7A2*-0.06588 + SCN1B*0.24403 + RGS16*0.16125 + TNFRSF9*-0.10820 + PDE4B*-0.40763 + NPFFR2*0.08028 + OSM*0.10854 + ROS1*-0.12802. The results, based on the median value, were used to distinguish between high- and low-risk groups (Figure 4G).

### 3.4 Multivariate cox prognostic regression model validation

**FIGURE 5:**
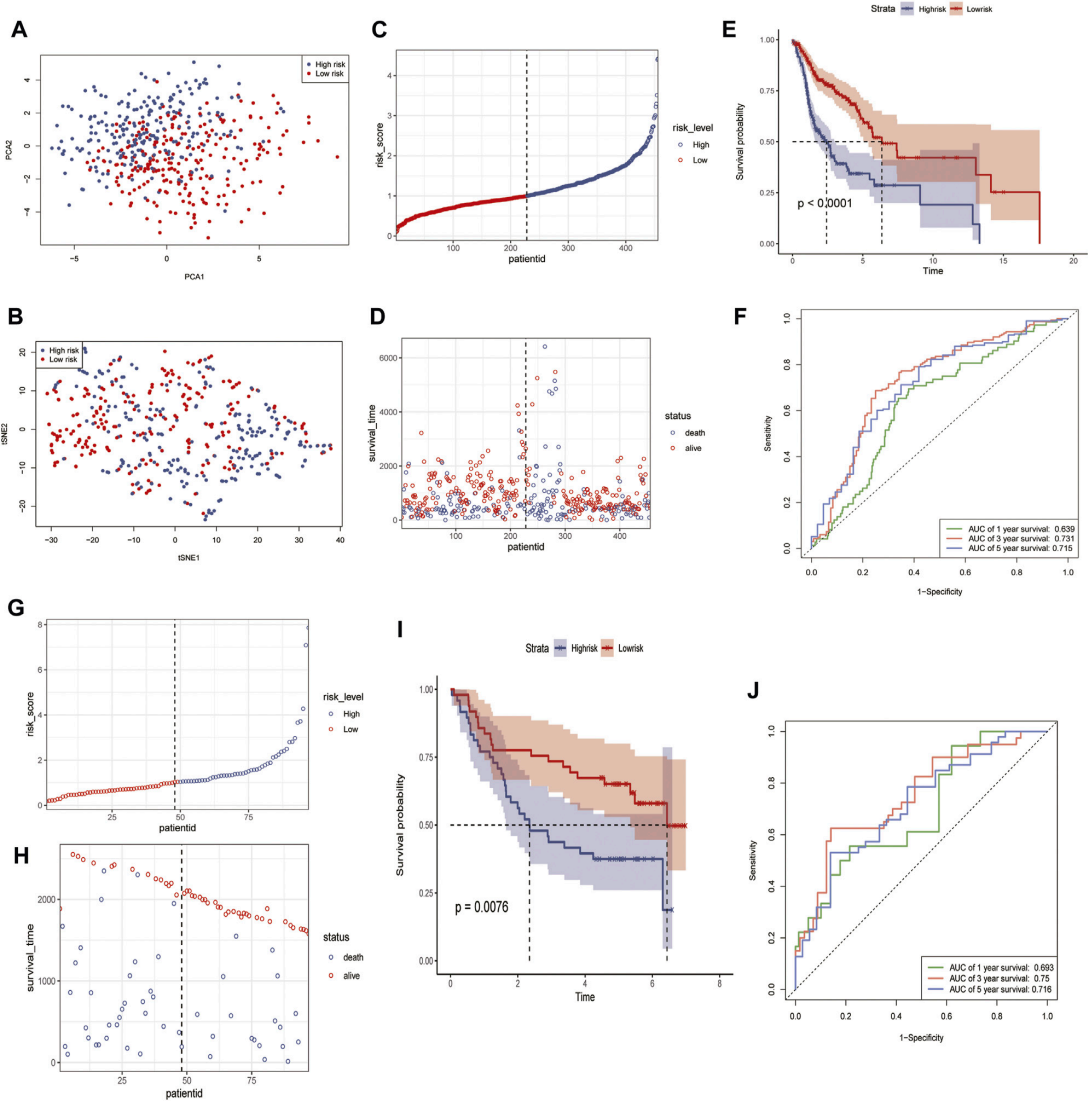
Identification and validation of 13 risk marker genes for prognosis in the TCGA cohort and GSE41613 using R software. **(A)** PCA in the TCGA cohort. **(B)** tSNE analysis of the TCGA cohort. **(C,G)** Risk score distribution in TCGA cohort **(C)** and GSE41613 **(G)**. **(D,H)** Distribution of survival time and survival status in TCGA cohort **(D)** and GSE41613 **(H)**. **(E,I)** Kaplan-Meier curves for the probability of overall survival in the risk group in TCGA **(E)** and GSE30219 **(I)**. **(F,J)** AUC curves for the three groups in TCGA **(F)** and GSE41613 **(J)**.

The PCA and t-SNE plots showed that the model sufficiently differentiated between the high- and low-risk groups (Figures 5A, B). The reliability of the IRGs in the independent cohort was tested by dividing TCGA cohort into low- and high-risk groups based on the cut-off values (Figure 5C). The KM curves showed significant differences in prognosis between the high- and low-risk groups; ROC curves, calculated using the timeROC R package, were used to assess survival prediction. The areas under the curve (AUC) were used to measure prognosis or predictive accuracy. The results showed that patients in the high-risk group were worse off than those in the low-risk group and had shorter survival times than those in the low-risk group (Figures 5D, E), with AUC values of 0.639, 0.731, and 0.715 at 1, 3, and 5 years, respectively (Figure 5F). Model validation was performed using the GSE41613 cohort as the validation set, and the high- and low-risk groups were distinguished using the median risk score (Figure 5G). Consistent with previous results for TCGA cohort, patients in the GSE41613 cohort were worse off in the high-risk group than in the low-risk group and had shorter survival times than those in the low-risk group (Figures 5H, I), with AUC values of 0.693, 0.75, and 0.716 at 1, 3, and 5 years, respectively, indicating that the risk score can be used to reliably predict the prognosis of patients with HNSCC (Figure 5J).

### 3.5 Enrichment analysis

**FIGURE 6:**
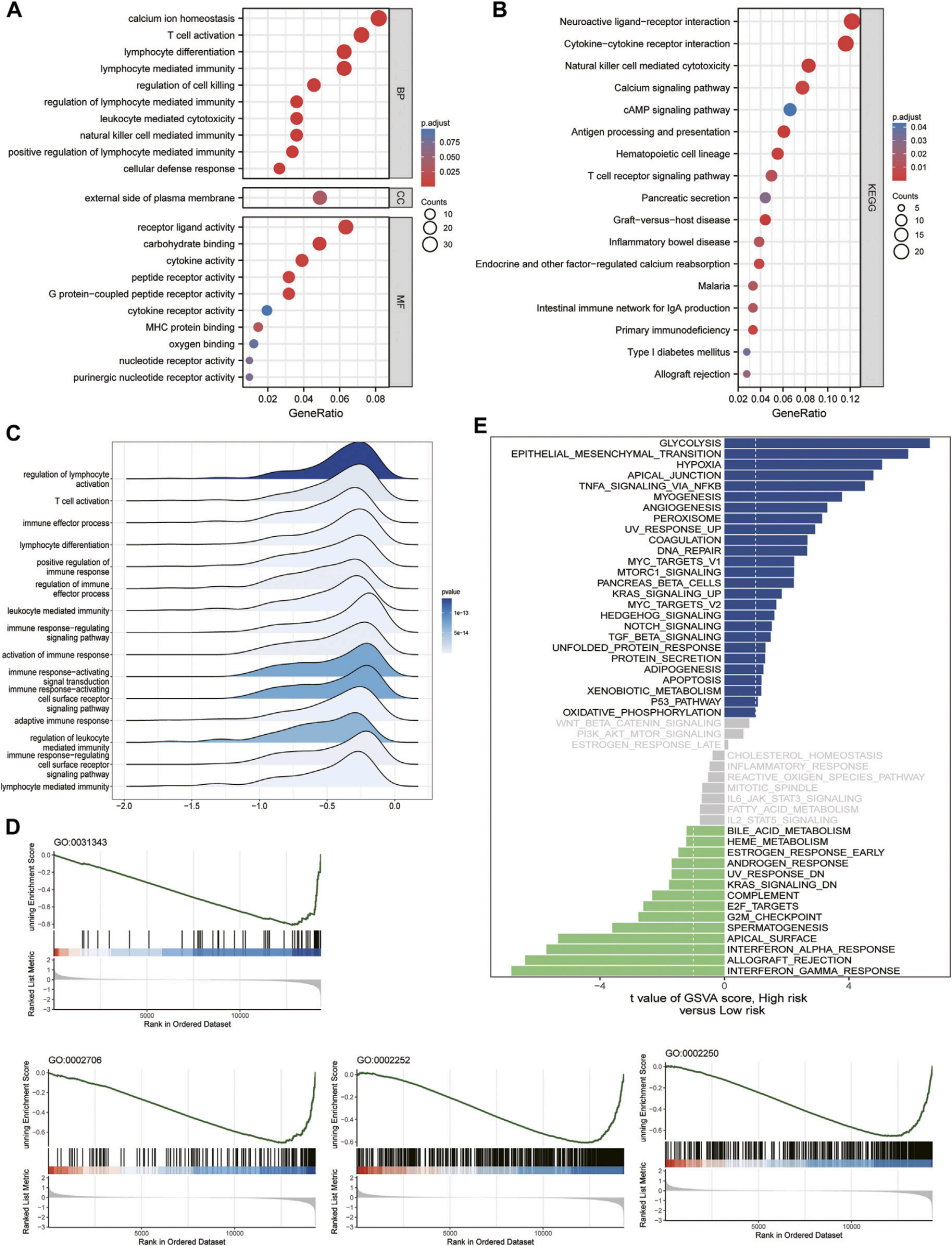
Functional enrichment analysis with GSE41613 cohort risk score grouping. **(A)** GO signaling pathway analysis. **(B)** KEGG signaling pathway analysis. **(C)** Ridge plot. **(D,E)** GSEA enrichment analysis and GSVA enrichment analysis.

We then performed GO, KEGG, GSEA, and GSVA functional enrichment analyses between the high- and low-risk groups to identify the significantly enriched activation pathways. As shown in the figure, the results indicated that the upregulated DEG in the high-risk group compared to the low-risk group, GO BP, was mainly enriched in calcium ion homeostasis, T Cell activation, and cytokines. In contrast, GO MF was mainly enriched in receptor ligand activity, carbohydrate binding, and cytokine activity (Figure 6A). The KEGG pathway analysis revealed that the main classifications were neuroactive ligand-receptor interaction, cytokine-cytokine receptor interaction, and natural killer cell-mediated cytotoxicity (Figure 6B). The above details were found in Supplementary Table S2. The ridge plot, GSEA, and GSVA enrichment analyses showed that the main differences between the high- and low-risk groups were immune-related (Figures 6C–E), which was consistent with previous results, the details were found in Supplementary Table S3.

### 3.6 Evaluation of the microenvironment

**FIGURE 7:**
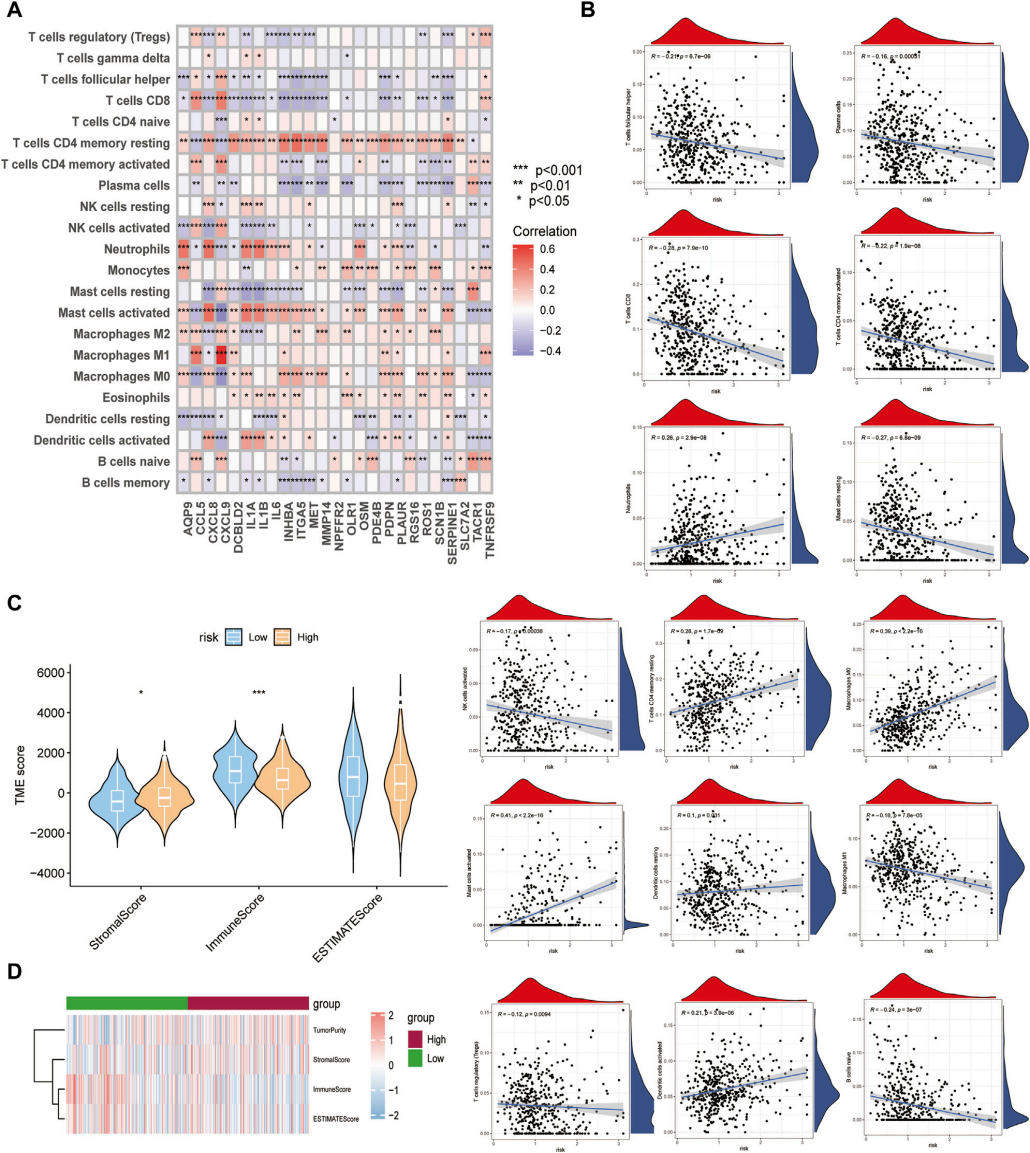
Differences in immune function between high- and low-risk subgroups. **(A)** Relationship between crucial cluster genes and immune infiltration. **(B)** Relationship between various types of immune cells and risk scores. **(C,D)** Comparison of Stromal, Immune, Estimate scores between high- and low-risk groups.

Although the results of this study can predict the prognosis for HNSCC patients, they are based on patient populations, which means that they cannot accurately predict the immune activation status of individual patients. Therefore, we performed a CIBERSORTx immune infiltration analysis and uploaded the gene expression matrix data (TPM) to CIBERSORTx. We filtered the output *p* < 0.05 samples to produce an immune cell-infiltration matrix. The results showed that the differential gene expression levels of the 25 relevant inflammatory factors were associated with immune function (Figure 7A) and the risk scores increased. The T_cells_CD4_memory_resting, Macrophages_M0, Mast_cells_activated, and Dendritic_cells_activated expression levels were elevated, whereas T_cells_follicular_helper, T_cells_CD8, T_cells_CD4_memory_activated, Macrophages_M1, T_cells_regulatory_(Tregs), and B_cells_naive decreased (Figure 7B), suggesting a decrease in specific immune function activation in tumor tissue with increasing risk score (Figures 7C, D), which correlates with a poor prognosis.

### 3.7 Drug sensitivity relationship with the multivariate cox prognostic regression model

**FIGURE 8:**
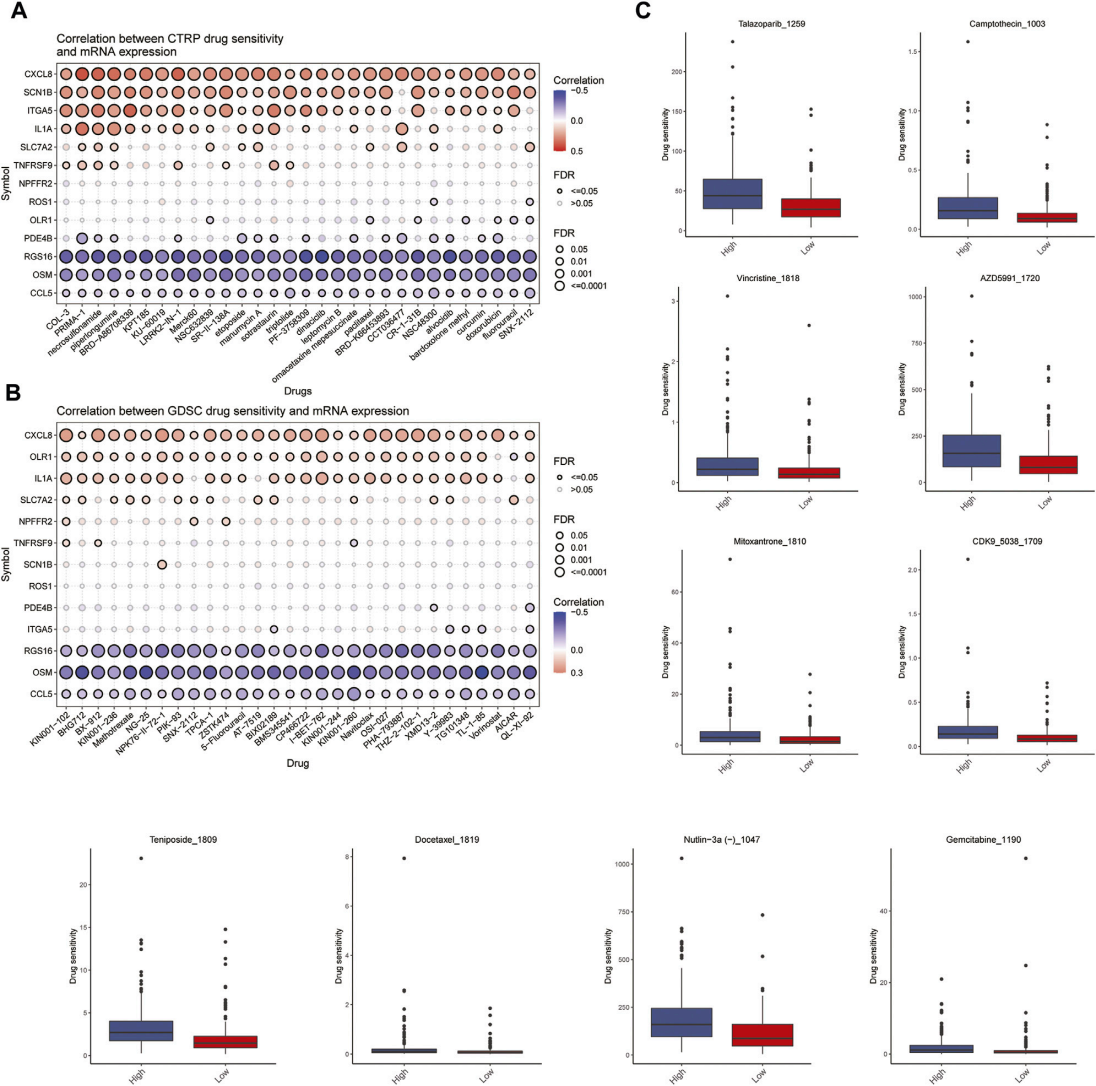
Analysis of drug sensitivity. **(A,B)** Relationship between Spearman correlation analysis (GSDC and CTRP) of 13 predicted model genes for drug sensitivity. **(C)** Differences in drug sensitivity between high and low risk groups of patients.

The relationship between model genes and drug sensitivity (GSDC and CTRP) was analyzed using the GSCALite database (http://bioinfo.life.hust.edu.cn/GSCA) (Figures 8A, B). In addition, drug sensitivity was analyzed using the oncoPredict package to compare the drug sensitivity of patients in the high- and low-risk groups. The results showed that patients in the high-risk group were more sensitive to talazoparib-1259, Camptothecin-1003, Vincristine-1818, Azd5991-1720, Teniposide-1809 and Nutlin-3a (-) −1047.Mitoxantrone-1810, Cdk9-5038-1709, Docetaxel-1819, and Gemcitabine-1190 were not significantly different between patients in the high and low risk groups. (Figure 8C).

### 3.8 *In vitro* validation

qRT-PCR analysis was used to examine the transcription levels of these genes in the tissues. The SCN1B and FDE4B expression levels are shown in Supplementary Figure S1. The SCN1B transcription level increased in laryngeal cancer tissues, whereas FDE4B transcription levels decreased. OLR1 showed no significant difference. This may be related to the small number of samples tested and the fact that we validated the tissue as a single laryngeal cancer.

## 4 Discussion

HNSCC is a tumor type that poses a severe threat to human health as most of the early symptoms are not significant and most patients are already in the middle to late stages when detected. This means that the 5-year survival rate is still below 65% (Miller et al., 2016). Therefore, identifying potential biomarkers and elucidating the molecular mechanisms underlying their development can improve tumor early diagnosis and prognosis. Existing bioinformatic analyses can be powerful tools for identifying biomarkers and therapeutic targets relevant to tumor progression and treatment. Previous studies have shown that novel serum biomarkers, including circulating tumor cells (Bates et al., 2018) and circulating nucleic acids (Tao et al., 2021), are good predictors of HNSCC prognosis. In addition, inflammatory response-related serum biomarkers, such as the neutrophil ratio, platelet-lymph-like ratio, and lymph-monocyte ratio, are also good predictors of HNSCC prognosis (Millrud et al., 2012). However, the use of inflammatory response-related genetic markers as prognostic markers for HNSCC has not been reported.

In recent years, changes in inflammation-related factors have been found to play a crucial role in regulating the progression of various cancers and can influence disease prognosis. Zhao et al. (2021) showed that inflammation-related genes are directly associated with immune infiltration and can improve prognosis prediction in gastric cancer (Zhao et al., 2021), which makes them a promising strategy for cancer treatment. Many studies have shown that inflammation-related factors play a crucial role as emerging genetic and molecular biomarkers in the biology of HNSCC (Lu and Jia, 2022). However, there have been no studies on the relationship between inflammatory factor-related genes and HNSCC prognosis. Based on the above research background, we investigated how genes encoding inflammation-related factors regulate the immune process, and thus, influence tumor progression and the prognosis for HNSCC patients.

Bioinformatic analyses were performed on normal skin and HNSCC samples based on TCGA and GSE41613 datasets. In this study, a differential analysis was performed on tumor samples taken from TCGA-HNSCC and standard samples. A total of 10,274 differential genes were obtained from the tumor samples and 57 differentially expressed IRGs were obtained by intersecting the differential inflammatory factor-related genes. We further performed a single-gene Cox regression analysis, and a total of 25 differential genes were obtained. The associated inflammatory factor differential genes were used for subsequent prognostic model construction. The 57 differentially expressed IRGs were used to divide 505 HNSCC patients into three different subgroups. Heatmaps linking gene expression levels and clinicopathological features among the three subgroups showed that there were significant differences, with survival curves indicating significant differences in OS between the two groups (*p* < 0.0001). Cluster 2 group had a significantly worse prognosis, suggesting that clinical classification based on differentially expressed IRGs could be used to predict patient prognosis. The functional enrichment results for the three subgroups showed that there were significant differences among the three cluster subgroups and these were mainly focused on immune-related pathways. The degree of immune cell infiltration was assessed using ssGSEA. Box plots were drawn to demonstrate the immune profiles of the different subclasses with the results showing that Cluster 1 and Cluster 3 had more robust activation of immune function than Cluster 2. This suggested that immune function may correlate with prognosis, with increased immune activation improving the prognosis for patients with HNSCC. Using univariate, LASSO, and multivariate cox regression analyses, we developed a prognostic risk model for HNSCC based on 13 genes associated with inflammatory factors (ITGA5, OLR1, CCL5, CXCL8, IL1A, SLC7A2, SCN1B, RGS16, TNFRSF9, PDE4B, NPFFR2, OSM, ROS1).The results were consistent with those of previous studies showing that CXCL1, CXCL2, CXCL3, CXCL8, and CXCL12 can be used as prognostic markers and potential therapeutic targets for patients with HNSCC (Jiang et al., 2020). Tian et al. (2020) showed that high expression of RGS16, LYVE1, snRNPs, ANP32A, and AIMP1 promotes cell proliferation and tumor progression, which are associated with the risk of death (Tian et al., 2020). Han et al. (2021) successfully developed a prognostic model consisting of COL4A1, PLAU, and ITGA5, and a survival analysis showed that the prognostic model could robustly predict OS (Han et al., 2021). The OLR1 gene mainly encodes a low-density lipoprotein receptor, and it has been shown that OLR1 promotes pancreatic cancer metastasis by increasing c-Myc expression and HMGA2 transcription (Yang et al., 2020). In addition, ORL1 also plays a role as a prognostic gene in the prognostic model of head and neck squamous cell carcinoma based on lipid metabolism-related genes (Gao et al., 2021). It has been shown that PDE4B in our prognostic model gene induces epithelial-mesenchymal transition in bladder cancer cells and is transcriptionally repressed by CBX7 (Huang et al., 2021). These results suggest that our prognostic genes play an important role in cancer, demonstrating that our model is of interest.The PCA and t-SNE plots showed that the model differentiated sufficiently between high- and low-risk groups. The patients in TCGA cohort were classified into low- and high-risk groups based on cut-off values, and survival prediction was assessed using KM and ROC curves. The results showed that patients in the high-risk group were worse off and had shorter survival times than those in the low-risk group. Furthermore, when the model was validated using the GSE41613 dataset as the validation set, the results similarly suggested that differentiating the high- and low-risk groups by the median risk score can be used to predict the prognosis of HNSCC patients.

Then, we performed a functional enrichment analysis between the high- and low-risk groups. The results showed that DEGs and GO BP were mainly enriched in calcium ion homeostasis, T Cell activation, and cytokines in the high-risk group, compared to the low-risk group. Membrane GO MF was mainly enriched in receptor ligand activity, carbohydrate binding, and the following cytokine activity KEGG pathway: neuroactive ligand-receptor interaction. These results suggest that the main differences between the high- and low-risk groups are immune-related, which was consistent with previous results (Yuan et al., 2022b; Konuthula et al., 2022). Therefore, we further performed a CIBERSORT immune infiltration analysis, which showed that the differential gene expression levels of the 25 relevant inflammatory factors were associated with immune function and increased the risk scores. T_cells_CD4_memory_resting, Macrophages_M0, Mast_cells_activated, and Dendritic_cells_activated expression levels were elevated. At the same time, T_cells_follicular_helper, T_cells_CD8, T_cells_CD4_memory_activated, Macrophages_M1, T_cells_ regulatory_(Tregs), and B_cells_naive decreased, suggesting that when the risk scores are higher, specific immune function activation in the tumor tissue is reduced and that this is associated with a poor prognosis (Taverna and Franchi, 2022). The relationship between the model genes and drug sensitivity (GSDC and CTRP) was analyzed using the GSCALite database. Our study showed that CXCL8, SCN1B, ITGA5, and IL1A were more sensitive to drugs, whereas RGS16, OS, and CCL5 were resistant to most drugs. We also screened for susceptibility between the high-risk and low-risk groups and showed that patients in the high-risk group were more sensitive to talazoparib-1259, camptothecin-1003, vincristine-1818, Azd5991-1720, Teniposide-1809, and Nutlin-3a (-) −1047.Finally, we examined the expression of OLR1, SCN1B and PDE4B genes in HNSCC pathological tissues and validated that these genes could be used to predict the prognosis of HNSCC.

Our experiments still have some limitations. First, due to missing data, we may have wider thresholds for the analysis process, so our prognostic model may reduce its applicability. Second, we need further *in vitro* validation of the role of genes in head and neck squamous cell carcinoma in prognostic models. Finally, because conditions are limited, although we performed pathological tissue validation for three genes in the model gene, it is better to perform further validation for all genes.

## 5 Conclusion

This study investigated the predictive role of immune pathways in the prognosis of HNSCC. We constructed a prognostic prediction model for HNSCC based on immune pathway genes using data related to HNSCC patients in TCGA database. The results from this study have the potential to help improve the diagnosis and treatment strategies for HNSCC.

## Data Availability

The original contributions presented in the study are included in the article/Supplementary Material, further inquiries can be directed to the corresponding author.
