# Supplementary material for: Effects of immune inflammation in head and neck squamous cell carcinoma: Tumor microenvironment, drug resistance, and clinical outcomes
Source: Front Genet. 2022 Dec 12;13:1085700. doi: 10.3389/fgene.2022.1085700 (PMC9790931; doi:10.3389/fgene.2022.1085700)
Supplement: Supplementary file 2 [file Table2.DOCX]

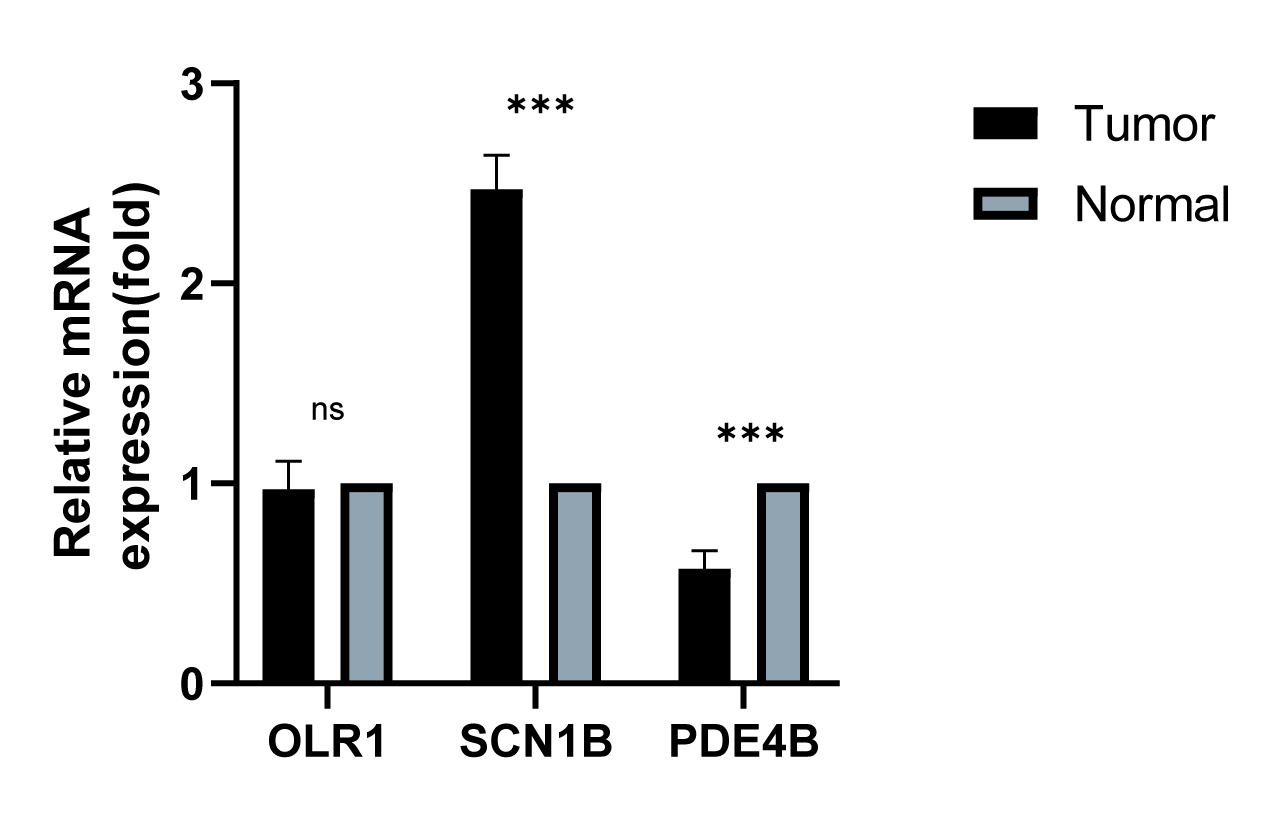


Figure S1|The expression level of prognostic genes.

All rawdata is in the Nut cloud：https://www.jianguoyun.com/p/Db3rOlAQh7PNCRj7tOIEIAA .
